# Cisplatin Induces Overactivation of the Dormant Primordial Follicle through PTEN/AKT/FOXO3a Pathway which Leads to Loss of Ovarian Reserve in Mice

**DOI:** 10.1371/journal.pone.0144245

**Published:** 2015-12-14

**Authors:** Eun Mi Chang, Eunjin Lim, Sookyoung Yoon, Kyungah Jeong, Sijeong Bae, Dong Ryul Lee, Tae Ki Yoon, Youngsok Choi, Woo Sik Lee

**Affiliations:** 1 Department of Obstetrics and Gynecology, Fertility Center of CHA Gangnam Medical Center, CHA University, Seoul, Republic of Korea; 2 Department of Biomedical Science, CHA University, Sungnam-Si, Republic of Korea; 3 Department of Obstetrics and Gynecology, School of Medicine, Ewha Womans University, Seoul, Republic of Korea; China Agricultural University, CHINA

## Abstract

Cisplatin is a first-line chemotherapeutic agent for ovarian cancer that acts by promoting DNA cross links and adduct. However drug resistance and considerable side effects including reproductive toxicity remain a significant challenge. PTEN is well known as a tumor suppressor function which plays a fundamental role in the regulation of the cell cycle, apoptosis and development of cancer. At the same time PTEN has been revealed to be critically important for the maintenance of the primordial follicle pool. In this study, we investigated the role of PTEN/Akt/FOXO3 pathway in cisplatin-induced primordial follicle depletion. Cisplatin induced ovarian failure mouse model was used to evaluate how this pathway involves. In vitro maturation was used for oocyte rescue after cisplatin damage. We found that cisplatin treatment decreased PTEN levels, leading to a subsequent increase in the phosphorylation of key molecules in the pathway. The activation of the PTEN/Akt/FOXO3 pathway cascade increased cytoplasmic translocation of FOXO3a in cisplatin-treated follicles, which in turn increased the pool size of growing follicles, and rapidly depleted the number of dormant follicles. Once activated, the follicles were more prone to apoptosis, and their cumulus cells showed a loss of luteinizing hormone (LH) receptor expression, which leads to failure during final maturation and ovulation. In vitro maturation to rescue oocytes in a cisplatin-treated mouse model resulted in successful maturation and fertilization. This study is the first to show the involvement of the PTEN/Akt/FOXO3 pathway in premature ovarian failure after cisplatin treatment and the possibility of rescue through in vitro maturation.

## Introduction

While the existence of ovarian failure after chemotherapy is well established, the precise mechanism is unclear. Until recently, the possible explanations for chemotherapy-induced premature ovarian failure (POF) were oocyte and somatic-cell apoptosis and cortical fibrosis [1, 2]. A new emerging hypothesis proposes that increased activation of follicles from the resting pool after chemotherapy and the eventual premature "burnout" of the primordial follicle reserve can cause POF [3–5].

Oocyte-specific knockout models have revealed the involvement of several molecules in the control of primordial follicle activation, including phosphatase and tension homolog (PTEN)[6]. PTEN is a negative regulator of the phosphatidylinositol 3-kinase (PI3K)/AKT pathway and has been known since 1997 as a universal tumor suppressor gene [7, 8]. PTEN is inactivated or mutated in various human malignancies, and aberrant PTEN-mediated signal transduction in cancer cells can cause a refractory response to chemotherapy [9–13]. However PTEN deficiency in oocytes of primordial and primary follicles indicates that PTEN/Akt/FOXO3 signaling in oocytes is critically important for maintenance of the primordial follicle pool rather than cancer development [14]. A recent study using the Cre-loxP system in transgenic mice carrying zona pellucida 3 promoter-mediated Cre recombinase revealed premature activation of the primordial follicle pool following the oocyte-specific deletion of PTEN [14, 15], after which the entire primordial follicle pool became depleted in young-adult mice, resulting in POF [6, 14]. Together with the BMP/AMH/SMAD pathway [16, 17], modulations in PTEN/Akt/FOXO3 pathways can accelerate or decelerate the rate of exhaustion of the ovarian reserve, causing POF or an extended period of fertility, respectively.

Cisplatin, one of the most common chemotherapeutic drugs, kills cancer cells by inducing the formation of inter-strand and intra-strand DNA adducts [3, 18]. Cisplatin is categorized as a member of the intermediate gonadal risk group of drugs and has been reported to cause ovarian failure with an odds ratio of 1.77 [19]. The mechanism of gonadal failure after cisplatin treatment is poorly understood. Recent evidence suggests that a nonreceptor tyrosine kinase called Abl is involved in cisplatin-induced cell death in early postnatal oocytes. Abl senses DNA damage, and when activated has a downstream effect on TAp63, a p53 homolog. The accumulation of Abl and Tap63 in oocytes eventually leads to cell death [20]. Additionally, cisplatin causes endoplasmic reticulum stress and induces the activation of the mitochondrial pathway leading to the caspase pathway activation [21, 22]. However, the precise mechanism of dormant primordial follicle loss is still undefined, and no definite apoptosis of primordial follicle and pregranulosa cells has been noted following cisplatin treatment [23]. A previous study that showed an association between PTEN blocking and primordial follicle outgrowth suggests that PTEN might be involved in POF following cisplatin treatment [13–15].

Here, we provide evidence that the PTEN/Akt/FOXO3a pathway is involved in cisplatin-induced primordial follicle depletion. Cisplatin treatment leads to a decrease in PTEN levels, resulting in the increased phosphorylation of AKT and enhanced FOXO3a extra-nucleation followed by an increase inthe multiple, simultaneous, activation of primordial follicles. Pan activation of dormant primordial follicles leads to an increased number of growing follicles, which are more susceptible to the apoptotic process and especially to luteinizing hormone(LH) receptor destruction. Furthermore, the in vitro maturation (IVM) of cisplatin-treated mouse oocytes during the outgrowth phase substantially improved the maturation and fertilization rates of the oocytes, providing the first possibility for oocyte rescue during cisplatin treatment.

## Materials and Methods

### Animal experiments

Six-week-old Slc:ICR female mice (n = 100) were purchased from Samtako (Seoul, Korea). The animals were housed in specific, pathogen-free conditions and allowed free access to a commercial rodent diet and tap water. The animal room was maintained at 20–26°C and 35–75% relative humidity with a 12 h light/dark cycle. After 5 days of quarantine, the mice were given daily intraperitoneal injections of various doses of cisplatin (Dong A pharmaceutical, Korea). The cisplatin was administered intraperitoneally once daily at doses of 0.5, 1.0, 1.5, and 2.0 mg/kg for 5 to 14 days. The dosing volume was 0.1ml for each injection. Ovary samples were collected at days 0, 5, 10, 12, and 15 after the first injection. Control animals received injections of 0.1ml phosphate buffered saline(PBS [Invitrogen, UK]). The animals were observed daily for clinical signs including weight loss, food intake and motor function. The body weight of each mouse was checked on the day of the first injection and on the day of ovarian tissue sampling. This study was carried out in strict accordance with the recommendations in the Guide for the Care and Use of Laboratory Animals of the National Institutes of Health. The protocol was approved by the CHA University Institutional Review Board for the conduct of research on animals (IACUC130021). We used humane endpoint for this study and endpoint was defined as more than 20% decrease in normal body weight, inability to reach food or water for more than 2 days, more than 7 days of 40% decreased appetite, continuous motor dysfunction including lethargy. Euthanasia was performed using cervical dislocation under tribromoethanol(Avertin) anesthesia, and all efforts were made to minimize suffering.

### Histology

#### Histological follicle assessment

The mouse ovaries were fixed in 4% paraformaldehyde (PFA), embedded in paraffin, serially sectioned (5μm thickness), and stained with hematoxylin for histology and differential follicle counting. For an accurate estimation of the size of primordial follicles, anti-LIM Homeobox 8 (Lhx8) (1:500, manufactured)[24], a molecular marker of early-stage follicles, was used for immunohistochemistry. Sections were incubated with blocking buffer (4% bovine serum albumin in PBS) containing 5% normal IgG for 1hr at room temperature and incubated with a primary antibody in blocking buffer for 1hr at room temperature and overnight at 4°C. The primary antibody was anti-Lhx8. After washing three times with PBS for 5mins, the sections were incubated with horseradish peroxidase-conjugated goat anti-guinea pig IgG as the secondary antibody (1:200, Invitrogen, UK) inblocking buffer for 2hrs at room temperature. Hematoxylin was used for nuclear counterstaining of the ovary sections. The sections were mounted on microscope slides (Fisher Scientific), and differential follicle counting was performed. Follicle counts were performed on representative sections from four mice from each group, and the mean count per section was calculated. Multiplication with a correction factor (20 = 5μm section thickness × the fraction of the ovary sampled) was done to obtain the total count [25]. The sections were put on a Nikon Coolscope Digital Microscope (Nikon, USA) for the differential follicle count. The follicle stage was classified according to previously accepted definitions [26]. A primordial follicle was defined as an oocyte surrounded by a single layer of flattened squamous pregranulosa cells. A primary follicle was defined as an oocyte surrounded by a single layer of cuboidal granulosa cells. Occasionally, follicles appeared as an intermediate stage between the primordial and primary stages, with both cuboidal and squamous granulosa cells. If the cuboidal cells predominated, the follicle was classified as a primary follicle. Secondary follicles had two or more layers of cuboidal granulosa cells with no visible antrum. Antral follicles were defined as follicles with an antral space containing follicular fluid. Preovulatory follicles were the largest follicles with a cumulous granulosa cell layer. For statistical analysis, the antral follicles and the preovulatory follicles were grouped together as antral follicles.

#### Terminal deoxynucleotidyl transferase-mediated dUTP-biotin nick end labeling (TUNEL) assay

After dewaxing, tissue sections were permeabilized with 10μg/ml proteinase K in 10mM Tris/HCl and then labeled with TUNEL reagents according to the instruction manual (Roche Diagnostics Ltd., Switzerland). The sections were counterstained with hematoxylin. The number of TUNEL-positive cells in the ovary was counted.

#### Immunofluorescence assay

The primary antibodies were anti-Lhx8 (1:500), anti-FOXO3 (1:100, Santa Cruz Biotechnology, USA), poly(ADP-ribose) polymerase (PARP)Antibody (Cell signaling, USA), and Antibody forLH-R (Acris antibodies Inc., USA). The secondary antibodies were Alexa 488 goat anti-rabbit and Alexa 546 goat anti-guinea pig (1:400, Invitrogen, UK). Actin and DAPI (Invitrogen, UK) were used to control the process of immune fluorescence. After staining, the sections were mounted. The staining was observed and images were obtained with a LSM 510 META confocal laser-scanning microscope (Carl Zeiss, Germany) or an epifluorescence microscope (Axio Imager 2, Carl Zeiss) with the ZEN image program.

#### Western analysis

Frozen ovaries were homogenized in a lysis buffer containing 150mM NaCl, 20mM Tris, 2% SDS, 1.7mM EDTA, 1.5mM sodium orthovanadate, 100mM NaF, 10mM sodium pyrophosphate, 17.5mM sodium β-glycerophosphate, 1.5μg/ml pepstatin, and protease inhibitor cocktail (Roche Diagnostics Ltd., Switzerland). The samples were heated to 95°C for 40 mins and centrifuged at 13000 rpm for 20 mins. The extracted protein samples were separated on SDS-PAGE gels, transferred to nitrocellulose membranes, and blocked with 5% non-fat milk in TBS containing 0.1% Tween 20 (TBST). The membranes were incubated with the following primary antibodies: anti-PTEN (1:1000, Millipore, USA), anti-AKT, anti-pAkt, anti-ERK, anti-pERK, anti-GSK3, anti-pGSK3β (1:1000, Cell Signaling, USA), and anti-α-tubulin (1:1000, Sigma-Aldrich, UK), diluted in TBST-5% milk at 4°C overnight. The appropriate horseradish peroxidase-conjugated secondary antibodies were diluted 1:1000 in TBST-5% milk and incubated for 1h at room temperature, and the signal was developed using the ECL Western Blotting substrate kit (Western-Q chemiluminescent substrate kit, Gendepot, USA).

### In vivo and in vitro maturation

B6D2F1 (Samtako, Seoul, Korea) were super-ovulated via intraperitoneal injections of 10IU pregnant mare’s serum gonadotropin (PMSG;Sigma), followed by 10IU human chorionic gonadotropin after 48hrs (hCG; Sigma). For the IVM of immature oocytes, immature germinal vesicle(GV) oocytes were collected 44–46hrs after the PMSG injection by puncturing the follicles with a 29G needle in Quinn’s Advantage Medium with HEPES (Quinn’s HEPES; Sage, in vitro fertilization, Trumbull, Inc., CT, USA) containing 10% substitute protein serum (SPS; Sage). The collected GV oocytes were cultured in TCM-199 containing 20% FBS, 0.075IU FSH (Gonal-F, Merck-Serono, Germany), 0.5 IU/ml hCG (Ovidrel, Serono), and 1.0μg/ml estradiol (Sigma-Aldrich) for 16hrs. The in vitro matured oocytes and ovulated mature oocytes were treated with 0.1% hyaluronidase to remove cumulus cells.

Epididymal spermatozoa were obtained from 10-week-old fertile B6D2F1 male mice. The sperm were capacitated for 60min and added to mature oocytes in Human Tubal Fluid(HTF) medium with 10% FBS. Six to eight hours later, fertilized oocytes with two pronuclei were collected and cultured in potassium simplex optimization medium(KSOM;Millipore, USA) containing 0.3% bovine serum albumin (BSA; Sigma-Aldrich). The fertilized oocytes were cultured in KSOM at 37°C in 5% CO_2_ for 6 days for analysis of embryonic development.

### Statistical analysis

All experiments were repeated at least three times. Statistical analysis was performed using IBM SPSS Statistics Version 20 (Statistical package for Social Science Japan, Inc., Tokyo, Japan). Quantitative variables are given as means ± standard error of the mean. The data were analyzed for statistical significance with the Student's *t*-test and the Mann-Whitney test. *P*-values <0.05 were considered statistically significant.

## Results

### The pool size of growing follicle increased during cisplatin treatment

**Fig 1:**
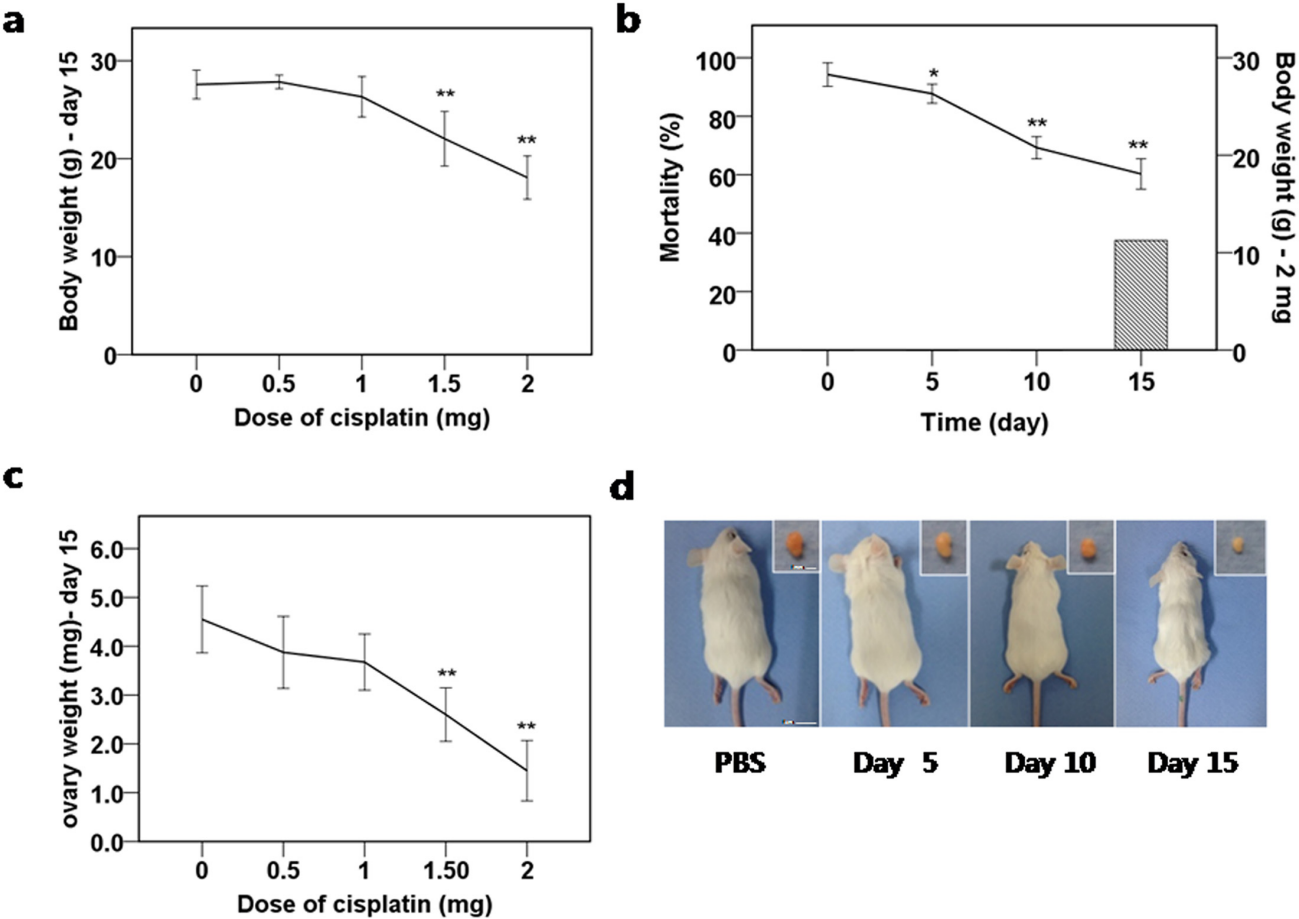
Body weight, mortality, ovarian weight, and gross morphology of the mice and ovaries after cisplatin daily injection. **(a)** Bodyweight of the mice after 15 days of daily injections with various doses of cisplatin. **(b)** Mortality (bar) and bodyweight (continuous line) of the mice receiving 2.0mg/kg daily cisplatin. **(c)** Ovarian weight after 15 days of daily injections with various doses of cisplatin. **(d)** Gross morphology of mice and ovaries after 2.0mg/kg daily injections with cisplatin (at 0, 5, 10, and 15 days starting from the left in the picture). **(a-c)** mean values ± SEM; stars denote significant differences relative to control (*P<0.05, ** P<0.01).

To study the mechanism of POF after cisplatin treatment, we first analyzed the dose effect of cisplatin on follicular growth in the mouse ovary. To find the optimal dose for causing POF after cisplatin treatment, mice were injected daily with 0.5mg, 1.0mg, 1.5mg, or 2.0 mg cisplatin per kg body weight for 14 days. The body weight, locomotor activity, and food consumption of the mice were monitored. The mice receiving 1.5mg/kg or 2.0mg/kg cisplatin lost significant weight and displayed decreased locomotor activity at day 15 (Fig 1a). The mice receiving 2mg/kg displayed significant weight loss and decreased locomotor activity starting from day 5, mortality occurred after day 15 (Fig 1b), and a time-dependent decrease in ovarian weight as the cumulative dose of cisplatin increased (Fig 1c and 1d). The mice receiving 1.5mg/kg had decreased ovarian weight only at day 15.

**Fig 2:**
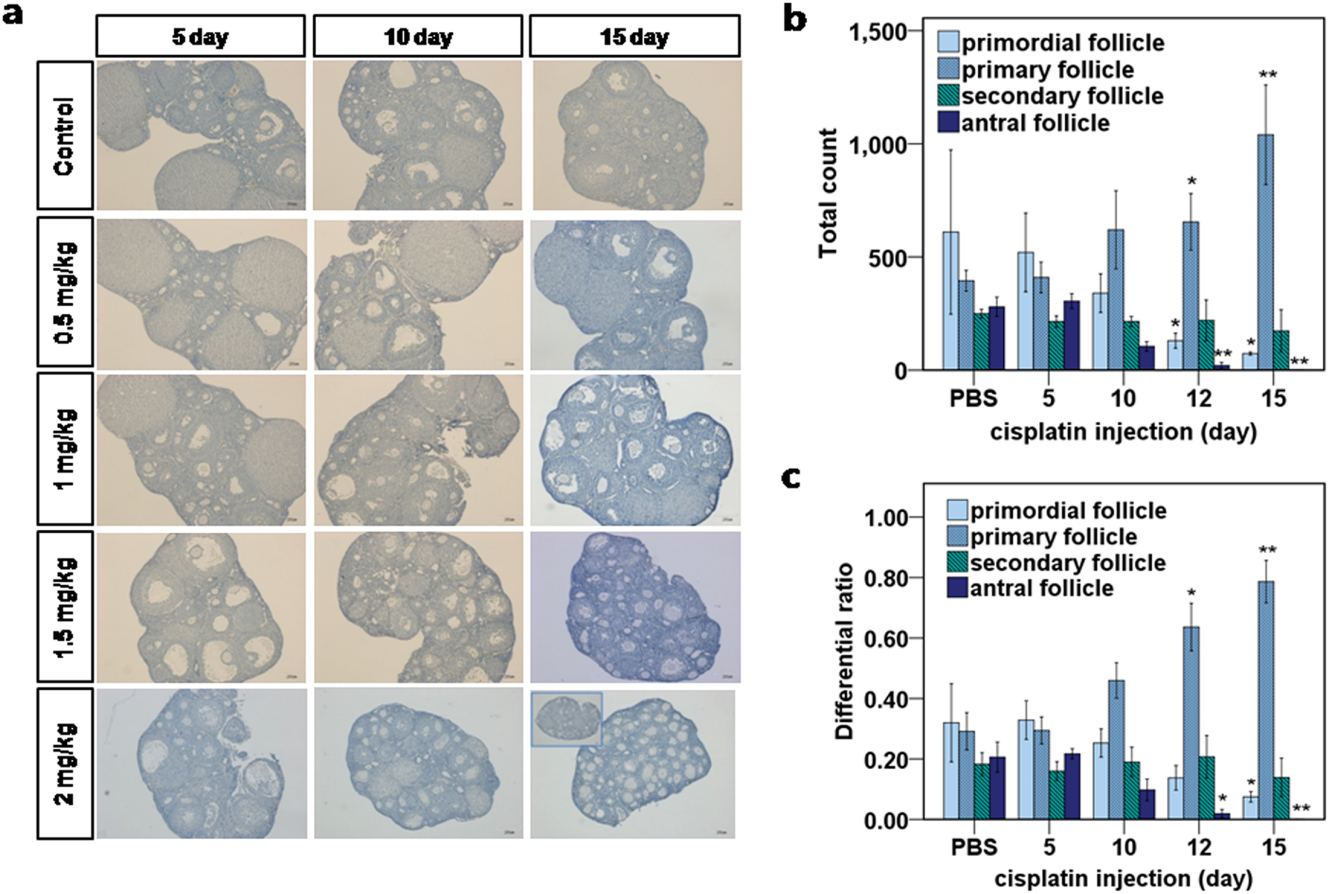
Representative histological sections of mouse ovaries at different time points after daily injection of various doses of cisplatin or PBS control. **(a)** Hematoxylin staining: Follicles at all stages were observed in control ovaries and in ovaries subjected to0.5mg/kg and 1.0mg/kg cisplatin. A significant decrease in the number of follicles beyond the antral stage was seen in the ovaries subjected to1.5mg/kg cisplatin after 15 days, and in those subjected to2.0mg/kg cisplatin after 12days(the inset at bottom right indicates day 12). An increase in the number of primary follicles was observed in the ovaries subjected to1.5mg/kg cisplatin after 15days, and in those subjected to2.0mg/kg cisplatin after 12 and 15 days. The primordial follicle depletion was definite after15 days of 2.0mg/kg cisplatin. **(b)** Differential counts of primordial, primary, secondary, and antral follicles and beyond. The total number of follicles at each stage and time point during 2.0mg/kg daily cisplatin treatment, revealed a significant increase in the total count of primary follicles after day 12. **(c)** The differential ratio of follicles at each stage and time point during cisplatin treatment showed a significant increase in the proportion of primary follicles after day 12. **(b-c)** mean values ± SEM; stars denote significant differences relative to control (*P <0.05, **P<0.01).

The histopathological findings for each group are shown in Fig 2a. The ovaries of the mice that received 0.5mg/kg or 1.0mg/kg cisplatin contained follicles in all stages of development (primordial, primary, secondary, antral, preovulatory, and corpus luteum) from day 5 to day 15. The ovaries of the mice that received 1.5mg/kg cisplatin contained increased numbers of primary follicles at day 15. The ovaries of the mice that received 2.0mg/kg cisplatin contained few preovulatory follicles, significantly decreased numbers of primordial follicles at day 10, and increased numbers of early growing follicles; at day 15, they contained almost no primordial preovulatory follicles or corpus luteum and increased numbers of primary and secondary follicles. Therefore, we concluded that 2.0mg/kg daily cisplatin injection for 15 days caused a clear change in follicle growth and thus constituted an adequate model of chemotherapy-induced POF. Furthermore, the cumulative human-equivalent dose for 2.0mg/kg daily injections over 5, 10, 12, and 15 days in mice (33, 66, 79.2, and 99 mg/m^2^, respectively) is within the therapeutic range of cisplatin doses in humans (40–140mg/m^2^). Therefore, we used the 2.0mg/kg daily injection model to quantify the follicles in response to cisplatin treatment and conduct further pathway analysis.

To improve our ability to count the follicles accurately, we immunostained the samples with anti-LIM Homeobox 8 (Lhx8) (1:500, manufactured)[24], a molecular marker of early-stage follicles. The quantification of the different follicle populations is shown in Figure A in S1 File. The numbers of primordial and mature follicles decreased as the number of treatment days increased (Fig 2b); however, the number of primary follicles increased significantly after day 12 (Fig 2b). When the differential ratio of each type of follicle was considered, the proportion of primary follicles increased significantly with the number of treatment days (Fig 2c). A comparison of the ratio of growing vs. nongrowing (dormant) follicles among time-points showed that the ratio increased over time and that significant increases occurred specifically at days 12 and 15(Figure B in S1 File). Few follicles progressed through the preovulatory stage. Rather, the vast majority of follicles became atretic and died during antral development.

### The PTEN/Akt/FOXO3 signaling pathway was activated after cisplatin treatment

**Fig 3:**
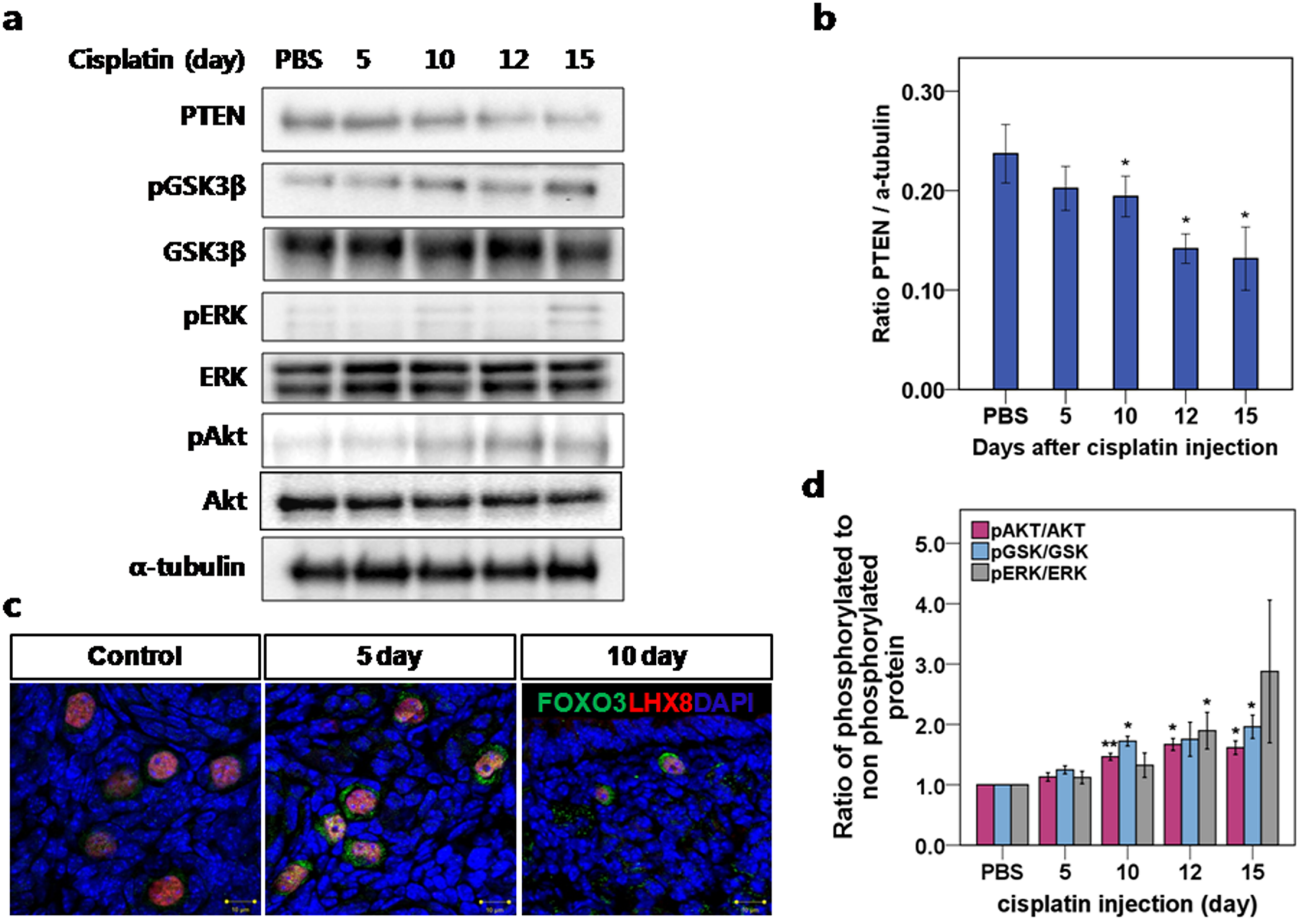
Cisplatin treatment triggered a decrease in PTEN levels and subsequent phosphorylation of key proteins in the PTEN/Akt/FOXO3 pathway. **(a)** Western blot of key proteins involved in the PTEN/Akt/FOXO3 pathway. **(b)** Protein analysis comparing the concentrations of PTEN with α-tubulin used as loading control. **(c)** Immunofluorescence staining demonstrated extra-nucleation (cytoplasmic localization) of FOXO3a in activated Lhx8-stained primordial follicles. In the control ovary, small follicles stained with Lhx8 showed nuclear localization of FOXO3a. After 5 and 10 days of daily 2.0mg/kg cisplatin injections, FOXO3a showed overall nuclear exclusion. **(d)** Protein analysis comparing the concentrations of phosphorylated and total AKT, ERK, and GSK3β, respectively. The ratio of phosphorylated to nonphosphorylated protein was calculated for each protein. **(b, d)** mean values ± SEM; stars denote significant differences relative to control (*P <0.05, **P<0.01).

Based on theobserved increase in the pool size of growing follicles, we analyzed the effect of cisplatin on the PTEN/Akt/FOXO3 signaling pathway. We removed whole ovaries from mice after 5, 10, 12, and 15 days of daily 2.0mg/kg cisplatin injections. We then analyzed the key proteins in the PTEN/Akt/FOXO3 pathway (PTEN, pGSK3β, GSK3β, pERK, ERK, pAKT, and AKT) using α-tubulin as a control. We detected a gradual decrease in PTEN levels until day 15 (Fig 3a and 3b), followed by the increased phosphorylation of key proteins in the PTEN/Akt/FOXO3 pathway (Fig 3a), which was apparent from the ratio between phosphorylated and nonphosphorylated proteins (Fig 3d). The increased phosphorylation of the proteins indicates PTEN/Akt/FOXO3 pathway activation, and previous studies have shown that the activation of that pathway is related to primordial follicle recruitment and exhaustion [14]. Those findings support the hypothesis that primordial follicle burnout is the mechanism of POF following cisplatin treatment. Schematic description of this hypothesis has been shown in Figure C in S1 File.

### Immunofluorescence staining revealed exonucleation of suppressor FOXO3a in primordial follicles

When FOXO3a proteins are phosphorylated and translocated from the nucleus, their transcription activities are suppressed. FOXO3a is highly expressed in oocyte nuclei in primordial follicles and is down regulated in the oocytes of primary and later growing follicles [27]. FOXO3a-knockout female mice exhibit global activation of primordial follicles within 2–3 weeks after birth and dramatic, age-dependent infertility due to POF [28, 29]. In control ovaries, we observed the nuclear localization of FOXO3a in small follicles stained with Lhx8; however, in ovaries treated daily for 5 or 10 days with 2mg/kg cisplatin, we observed the increased nuclear exclusion of FOXO3a. Ratios of follicle with FOXO3 nuclear exclusion and LHX8 positive cells at 0, 5 and 10 days were 0.28, 0.47 and 0.42, respectively. These findings supports the hypothesis that the activation of primordial follicles leads ultimately to follicle depletion and POF (Fig 3c).

### TUNEL staining revealed no evidence of the apoptotic loss of primordial follicles

**Fig 4:**
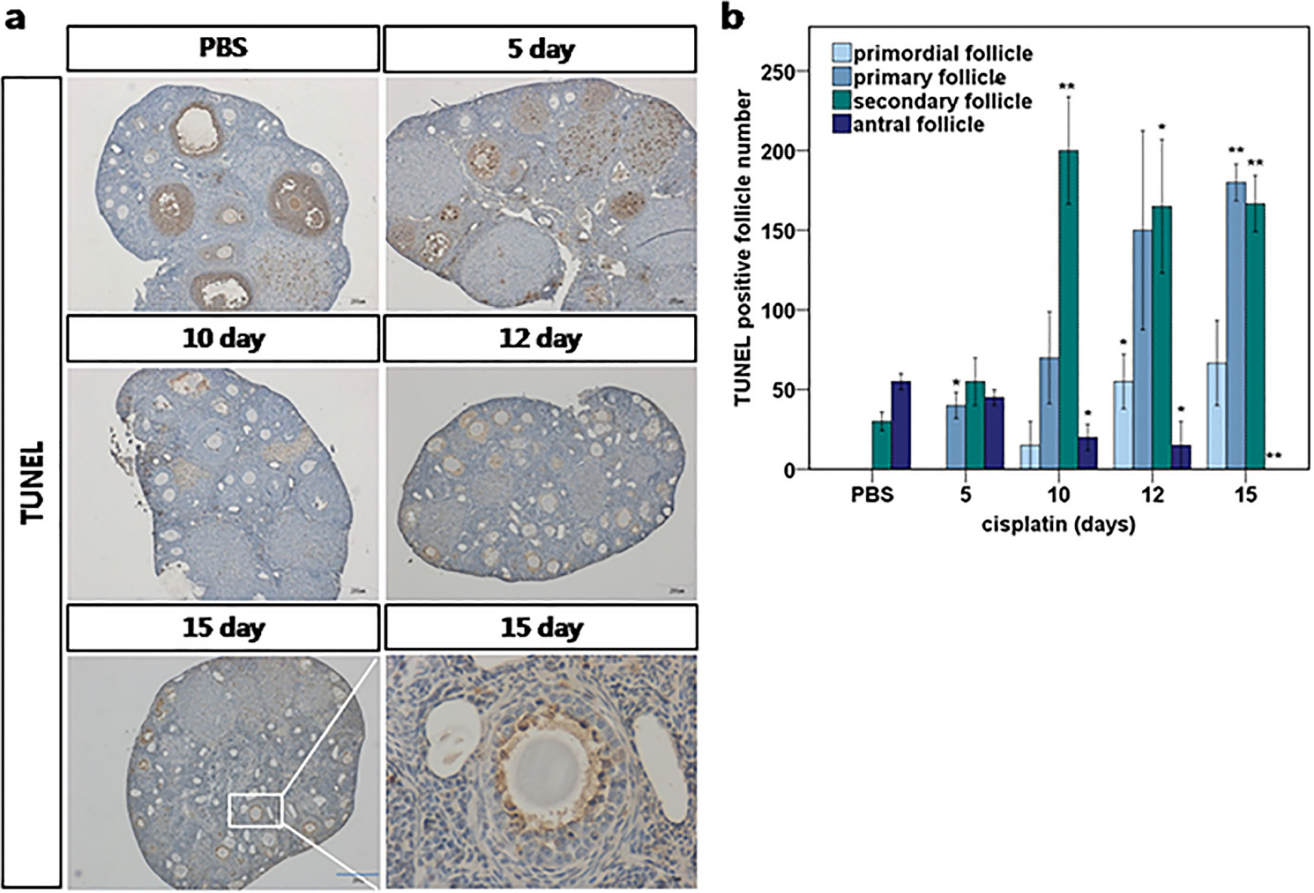
TUNEL staining of ovaries from cisplatin-injected mice. **(a)** Ovary tissues from mice treated with 2mg/kg daily cisplatin. The sections were stained with TUNEL and counterstained with hematoxylin at each time point. The granulosa cells of secondary follicles after 15 days of cisplatin injections are positive for TUNEL while the oocyte is protected. **(b)** The number of TUNEL-positive follicles for each stage at different time points. Data represent means ± SEM. Stars denote significant differences relative to control (*P <0.05, **P<0.01).

To determine whether apoptosis was involved in the primordial follicle loss, we performed TUNEL staining of the ovaries harvested after 0, 5, 10, 12, and 15 days of daily cisplatin treatment (Fig 4a). We observed TUNEL positivity at all time-points in preovulatory follicles and in the corpus luteum, mainly in the granulosa cells. TUNEL positivity in primordial follicles was scant, however, and decreased sharply between days 5 and 10. We mainly observed TUNEL positivity in primary and secondary follicles while the numbers of follicles at those stages remained steady or increased. Apoptosis was evident in the primordial follicles only when few primordial follicles remained after day 12(Fig 4b). Our observations were consistent with the prior understanding that cells are most susceptible to chemotherapeutic agents when they are rapidly proliferating. The results of TUNEL staining indicate that apoptosis increased among growing follicles due to the shift away from primordial follicles, rather than among them.

### Immunohistochemical staining of cleaved PARP revealed widespread apoptosis among the granulosa cells of secondary follicles

**Fig 5:**
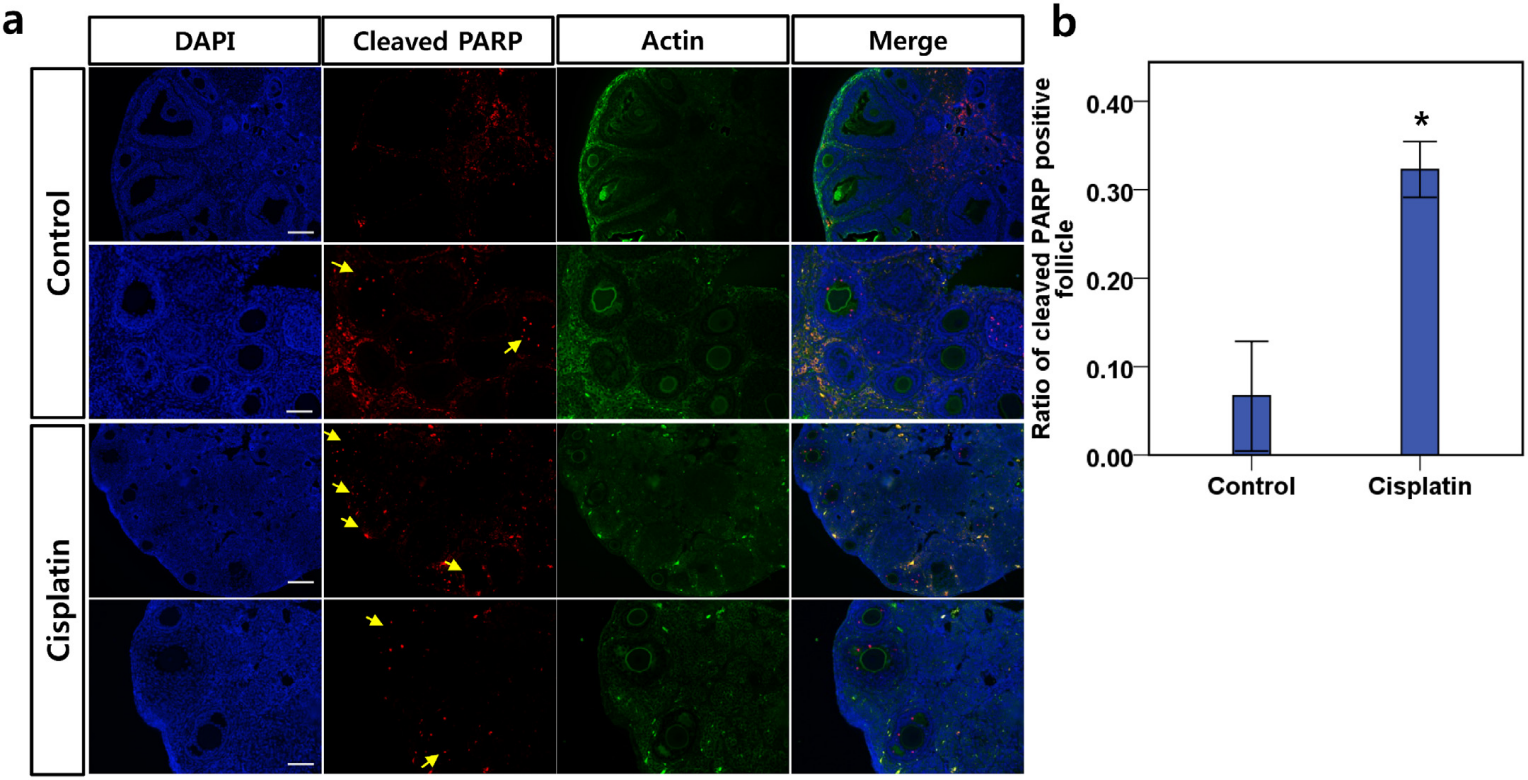
Cleaved PARP expression in secondary follicles. **(a)** Immunohistochemistry of ovarian tissue; cleaved PARP was observed in the granulosa cells of secondary follicles from cisplatin-treated mice. Cleaved PARP (red) counterstained with DAPI (blue) and actin (green). The arrow (yellow) indicates the cleaved PARP in secondary follicles. Scale bars = 100μm. **(b)** Ratio of cleaved PARP-positive follicles from PBS-injected control mice and cisplatin-treated mice. Data represent means ± SEM. Stars denote significant differences relative to control mice injected with PBS (*P <0.05)

To evaluate the presence of single-strand and double-strand DNA breakage in oocytes and follicular cells, we performed cleaved poly(ADP-ribose) polymerase (PARP; MW 113KDa) immunohistochemistry on mouse ovarian tissues after 10days of 2mg/kg daily cisplatin treatment (Fig 5). PARP is a DNA repair protein, and cleaved PARP (MW 89KDa) is a hallmark of apoptosis. Immunohistochemical staining of ovarian tissues detected cleaved PARP in the granulosa cells of secondary follicles from control and cisplatin-treated mice (Fig 5a). More than 32.3%of the secondary follicles from cisplatin-treated mice displayed cleaved PARP, whereas only 6.7% of the secondary follicles from control mice displayed cleaved PARP (Fig 5b).

### In vivo maturation failed due to LH receptor destruction

**Fig 6:**
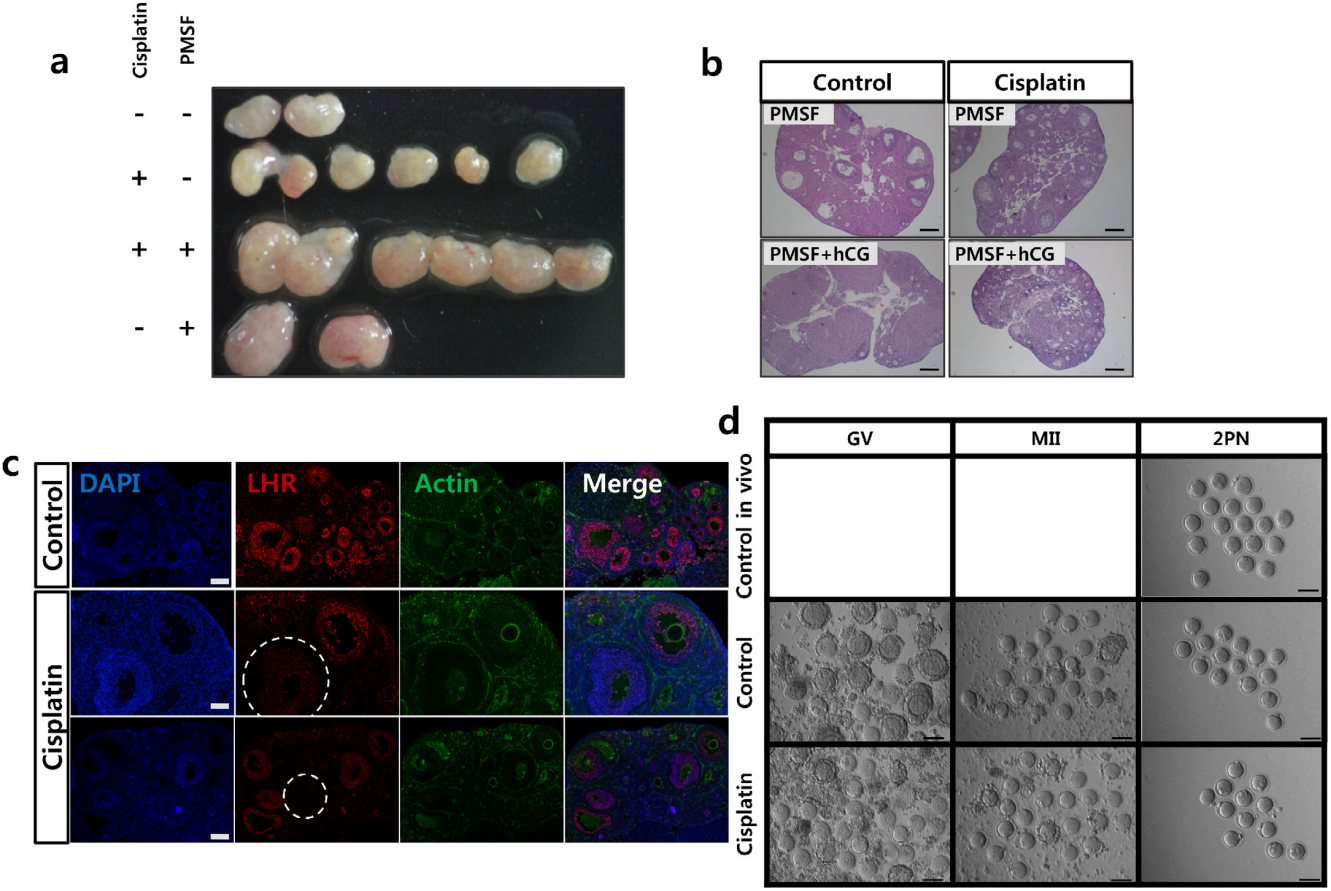
PMSG injection induced follicular growth but failed to induce ovulation in cisplatin-treated ovaries. Ovaries removed from ICR mice 48h after PMSG injection. **(a)** Comparison of the size of cisplatin-treated ovaries with or without PMSG. **(b)** Midline histological sections of ovaries removed after PMSG and hCG injections and stained with hematoxylin and eosin. The corpora lutea in the control ovary are shown after hCG injection. Scale bars = 100μm. **(c)** The expression of LH receptor was detected at 10 days after PBS or cisplatin injection. LH receptor (red) counterstained with DAPI(blue) and actin (green). In the control ovary, most of the follicles past the primary stage expressed LH receptor; however, half of the follicles from the cisplatin-treated mice did not express LH receptor(in circle). **(d)** In vivo and in vitro maturation of mouse oocytes after cisplatin treatment. Oocyte maturation (MII) and fertilization (two pronuclei, 2PN) of oocytes from ovaries after cisplatin treatment matured in vivo or in vitro. GV, germinal vesicle: MII. Scale bars = 100μm.

We observed ovaryenlargement due to multiple follicle development in vivo aftertreatment with PMSG (Fig 6a and 6b). The post-PMSG intraperitoneal injection of 10 IU human chorionic gonadotropin (hCG) inducedthe ovulation of three oocytes from a total of six ovaries in cisplatin-treated mice, and of 25 oocytes from a total of two ovaries in control mice. Histological sections of ovaries removed after the PMSG and hCG injections revealed increased numbers of growing follicles without visible corpus luteum in the cisplatin-treated mice, while corpora luteawere relatively prominent in the control ovaries after the hCG injection (Fig 6b). Because the final maturation of the dominant follicle and the health of the oocyte are optimized by the required presence of a threshold level of LH, we evaluated the expression of LH receptor in the ovaries. The expression of LH receptor was detected 10 days after daily PBS or cisplatin 2mg/kg treatment. In control mice, most of the follicles at developmental stages beyond that of the secondary follicle expressed LH receptor; however, there was little LH receptor expressed in the follicles from the cisplatin-treated mice (Fig 6c).

### In vitro maturation of cisplatin-treated oocytes improved maturation and fertilization rates

We next assessed the possibility of oocyte rescue after cisplatin treatment through IVM, which could avoid the possible hampering effect of granulosa cell(GC) apoptosis on successful ovulation. IVM was induced by PMSG injection of mice on day 10 of 2.0 mg/kg daily cisplatin treatment, the day when the increase in the pool size of growing follicles became evident. We removed the ovaries from cisplatin-treated mice 48h after the PMSG injection and assessed the competence of the oocytes and embryos after IVM and fertilization. The cisplatin/PMSG-treated ovaries produced immature oocytes and were larger than cisplatin/nonPMSG-treated ovaries, being comparable in size to the non cisplatin/PMSG-treated ovaries (Fig 6a and 6d). The GV oocytes collected from cisplatin-treated ovaries had few surrounding granulosa cells compared with control oocytes, and their granulosa cells were darkerand discolored (Fig 6d). There were no significant differences, however, in the maturation rate and the two-pronuclear zygote(2PN) formation rate after IVM (Table 1).

**Table 1:** Effect of cisplatin on oocyte maturation, fertilization, and embryonic development in vitro.

	Total number of ovaries	Number of oocytes per ovary	Total number of GV oocytes	MII (% ± SEM/GV oocytes)	MII oocytes for IVF	2PN (%/MII oocytes)
**Control**	**5**	**10.6**	**53**	**39(73±2%)**	**20**	**14(70%)**
**Cisplatin**	**11**	**4.8**	**53**	**39(75±5%)**	**15**	**10 (67%)**

Data are presented as the total numbers and average percentages ± SEM from two replicates for in vitro maturation, and one replicate for fertilization and embryonic development. GV, germinal vesicle; MII, oocytes arrested at the second meiotic division; BL, blastocyst; 2PN, two pronucleus. The MII ratio was calculated from GV immature oocytes cultured for 16h in vitro. 2PN was calculated from MII oocytes 7h after in vitro fertilization.

## Discussion

In the present study, we demonstrated that cisplatin treatment resulted in the premature exhaustion of primordial follicles, which could be induced by decreased PTEN levels, the sequential phosphorylation of AKT, and the nuclear export of FOXO3a. When primordial follicles are unleashed, their number decreases and the number of primary and secondary follicles grow, resulting in higher susceptibility of the follicles to apoptosis. Our IVM study showed that the follicles activated after cisplatin treatment were competent for maturation and fertilization.

Until recently, the main explanation for chemotherapy-induced POF was the direct apoptosis of primordial follicle cells [3, 30]. However there are a few evidence documenting primordial follicle apoptosis in vitro [31]. Theoretically, chemotherapy targets actively dividing cells. Therefore, the logical targets for chemotherapy are not dormant, mitotically inactive cells, but rather the proliferating somatic cells around growing or mature follicles. Additionally, a recent study demonstrated that cisplatin treatment does not increase the number of TUNEL-positive primordial cells [23]. In line with prior report, primordial follicle apoptosis was insignificant in our study. Instead, the excessive expenditure of primordial follicles was the cause of primordial follicle loss after cisplatin treatment. We observed that the number of primordial follicles decreased without indications of increased apoptosis, while the prevalence of apoptotic features in primary and secondary follicles increased with the duration of cisplatin treatment. Additionally, an analysis of cleaved PARP revealed that, for the most part, the granulosa cells in the pool of growing follicles beyond the secondary follicle stage were susceptible to cisplatin-induced DNA damage. Our results are inconsistent with the hypothesis that primordial follicle apoptosis is the mechanism of POF after cisplatin treatment.

Previous studies have shown that chemical agents can cause POF in rodent models by enhancing the PI3K and mTOR1 signaling pathways. The polycyclic aromatic hydrocarbon 7, 12-demethylbenz-(a)anthracene and 3-methylcholanthrene have been shown to induce ovotoxicity through the over-activation of primordial follicles by enhancing the activation of PI3K and mTORC1 signaling in a rodent model [32, 33]. A recent study showed that among the available chemotherapeutic agents, cyclophosphamide increases the phosphorylation of proteins involved in PI3K signaling, which stimulates follicle activation and leads to premature primordial follicle loss [5]. Our results concur with previous findings and we suggest PTEN as key regulator in cisplatin-induced POF. Although our study did not evaluate the reason for PTEN decrease after cisplatin treatment, evidence can be found in previous studies. Cisplatin induced caspase activation which leads to the post-translational attenuation of PTEN protein which results in AKT phosphorylation in ovarian cancer cells [11]. Additionally, cisplatin resistance in human ovarian cancer cells are related with aberrantmiR-21 expression which in turn mediates PTEN decrease [34].

Previous studies have shown that the over-activation of PI3K signaling in oocytes does not appear to affect the development of growing follicles [6, 14]. The use of a PTEN inhibitor and a PI3K-activating peptide was shown previously to increase the nuclear exclusion of FOXO3a in primordial oocytes and to activate primordial follicles to a stage where the follicles can respond to follicle-stimulating hormone(FSH)[35]. When ovarian cortical fragments from patients with cancer were treated with a PTEN inhibitor and transplanted into immune-deficient mice, primordial follicles developed to the preovulatory stage within 6 months, with oocytes capable of undergoing nuclear maturation [36]. In our study, however, after the activation of primordial follicles, the number of primary follicles increased, although the follicles failed to mature in vivo and almost none reached the antral stage. Our findings indicate that there are other obstacles in addition to PTEN/Akt/FOXO3 pathway activation to follicles reaching final maturation during cisplatin treatment. An extensive number of early growing follicles may not be optimally supported when the hypothalamus-pituitary-ovary axis is disrupted following cisplatin treatment. Alternatively, the apoptosis of granulosa cells, which is triggered by cisplatin treatment, may be one reason why the pool of growing follicles did not prolong follicle survival. The LH receptor was universally destroyed in follicle stages beyond that of secondary follicle and during final maturation in vivo, and the function of the follicle may be significantly altered in vivo after cisplatin treatment. Our IVM results show that primordial follicles activated after cisplatin treatment can successfully mature and become competent for fertilization in vitro. For further growth and development of in vitro fertilized embryos, diverse culture techniques are being developed.

We suggest further research direction toward modulating the PTEN/Akt/FOXO3a pathway during cisplatin treatment to suppress primordial follicle burnout and preserve ovarian function. Such a noninvasive method of preventing POF would be preferable to invasive methods for preserving fertility, such as gamete and embryo cryopreservation, which are unavailable to some patients who requireurgent chemotherapy. PTEN modulators have already been developed or are currently under development for use in various diseases including diabetes, cardiovascular disease, obesity, autism, Parkinson’s disease, and cancer. Monoclonal antibodies, peptides, or inhibitors that are specific for kinases, transcription factors, and cellular proteins affecting PTEN could all be candidates for ancillary treatments to rescue ovarian function during and after chemotherapy [37]. However, the use of PTEN modulators to prevent cisplatin-induced POF will require further supportive evidence. Such ancillary treatments must be shown, for example, to not interfere with the function of cisplatin as a cancer treatment. Agents to reduced chemoresistance by up regulating PTEN expression may affect fertililty reserve after cisplatin treatment and can be interesting issue to be co evaluated. An additional issue is the possibility of aneuploidy after chemotherapy, which raises concerns about pregnancy outcomes even if a sufficient pool of primordial follicles is preserved. In an animal study, cisplatin and its analogues caused aneuploidy in mature oocytes and were linked to high rates of embryonic mortality [38]. However, no observable increases in fetal malformation or miscarriage after anticancer treatment have been reported in humans [39].

We demonstrated that cisplatin over-activates dormant primordial follicles in mice, reducing the pool size of ovarian follicles. Cisplatin activated the PTEN/Akt/FOXO3 pathway, thus increasing the pool size of growing follicles while rapidly depleting the number of dormant follicles. Two additional factors that contributed to POF were the accelerated apoptosis among growing follicles, and a decrease in the number of LH receptors after cisplatin treatment. Our findings suggest a promising approach to fertility preservation during cisplatin treatment, as well as the possibility of oocyte rescue after cisplatin treatment through IVM. Because the mechanisms and toxicities of different chemotherapeutic agents are distinct, our findings may not be generalizable to all chemotherapeutic agents, and finding a specific solution for each chemotherapeutic agent will be another field of research.

## Supporting Information

S1 File Figure A. Representative Section from 2mg/kg injected mouse ovary were subsequently stained with Lhx 8. Figure B. Comparison of the ratio of growing vs. non-growing (dormant) follicles. Figure C. Proposed scheme illustrating the mechanism behind premature ovarian failure induced by cisplatin treatment.
(DOCX)
