# Supplementary material for: Cisplatin Induces Overactivation of the Dormant Primordial Follicle through PTEN/AKT/FOXO3a Pathway which Leads to Loss of Ovarian Reserve in Mice
Source: PLoS One. 2015 Dec 14;10(12):e0144245. doi: 10.1371/journal.pone.0144245 (PMC4699462; doi:10.1371/journal.pone.0144245)
Supplement: S1 File — Figure A. Representative Section from 2mg/kg injected mouse ovary were subsequently stained with Lhx 8. Figure B. Comparison of the ratio of growing vs. non-growing (dormant) follicles. Figure C. Proposed scheme illustrating the mechanism behind premature ovarian failure induced by cisplatin treatment. (DOCX) [file pone.0144245.s001.docx]

**
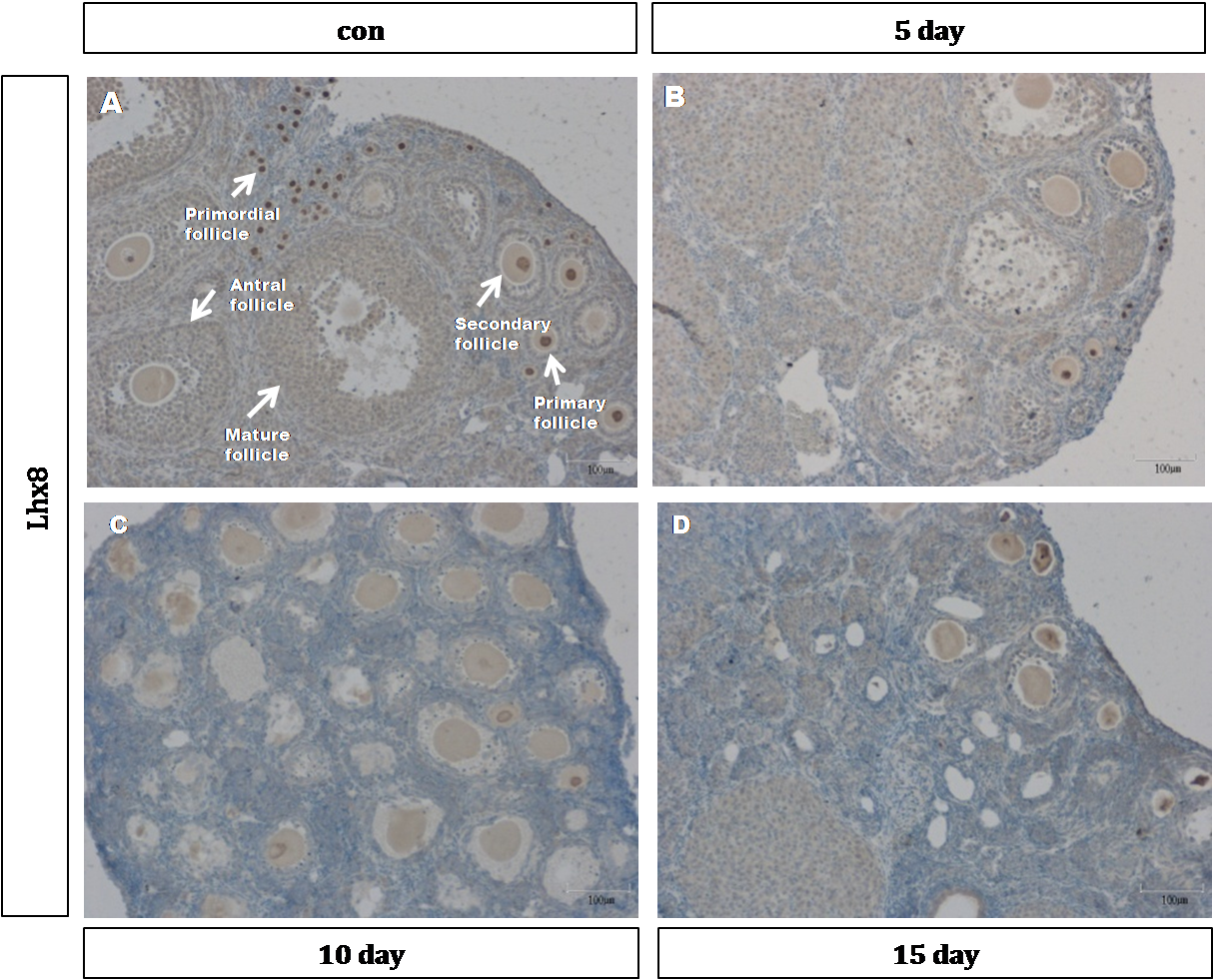
**

**Figure A.** **Representative Section from 2mg/kg injected mouse ovary were subsequently stained with Lhx 8.** Nest of oocytes from primordial follicle are well stained with Lhx8 at control ovary in A, while few detected after day 10 in C and D.

**Figure B. Comparison of the ratio of growing vs. non-growing (dormant) follicles**. Ratio between growing versus nongrowing follicles at each time point showed that the ratio increased at all time points and that a significant increase was noted at days 12 and 15.

*****

**Figure C. Proposed scheme illustrating the mechanism behind premature ovarian failure induced by cisplatin treatment.** As cisplatin accumulates and reaches a certain dose, the PTEN level decreases. The decrease in PTEN levelactivates the pathway. The subsequent full-blown growth of primordial follicles following thePTEN/Akt/FOXO3 pathway activation ultimately leads to apoptosis. As the growingpool of follicles, including oocytes and granulosa cells,ishighly susceptible to apoptosis, premature ovarian failure accelerates.
